# Emulative learning of a two-step task in free-ranging domestic pigs

**DOI:** 10.1007/s10071-022-01740-3

**Published:** 2023-01-18

**Authors:** Ariane Veit, Stefanie Weißhaupt, Arnaud Bruat, Marianne Wondrak, Ludwig Huber

**Affiliations:** Messerli Research Institute, University of Veterinary Medicine Vienna, University of Vienna, Medical University of Vienna, Veterinaerplatz 1, 1210 Vienna, Austria

**Keywords:** Social learning, Heterospecific, Ghost control, Emulation, Affordance, Sus scrofa

## Abstract

Previous research showed that young domestic pigs learn through observation of conspecifics by using social learning mechanisms like social facilitation, enhancement effects, and even object movement re-enactment. The latter suggests some form of emulative learning in which the observer learns about the object’s movements and affordances. As it remains unclear whether pigs need a social agent to learn about objects, we provided 36 free-ranging domestic pigs with varying degrees of social to non-social demonstrations on how to solve a two-step manipulative foraging task: observers watched either a conspecific or a human demonstrator, or self-moving objects ("ghost control"), or a ghost control accompanied by an inactive conspecific bystander. In addition, 22 subjects that were previously tested without any demonstrator were used as a non-observer control. To solve the task, the subjects had to first remove a plug from its recess to then be able to slide a cover to the side, which would lay open a food compartment. Observers interacted longer with the relevant objects (plugs) and were more successful in solving the task compared to non-observers. We found no differences with regard to success between the four observer groups, indicating that the pigs mainly learned about the apparatus rather than about the actions. As the only common feature of the different demonstrations was the movement of the plug and the cover, we conclude the observer pigs learned primarily by emulation, suggesting that social agents are not necessary for pigs when learning through observation.

**Supplementary Information:**

The online version contains supplementary material available at 10.1007/s10071-022-01740-3.

## Introduction

An important way to adapt to changing circumstances is to acquire new behaviours, either by individual trial-and-error learning or by learning through observing others. While individual learning can be costly in terms of time and energy spent (or potentially even fatal), observational or social learning might provide an already established shortcut to new behaviours (Boyd and Richerson 1988; Galef and Laland 2005; Huber 2012; Laland 2004; Zentall 2006). Observers may learn how to perform certain actions by copying the demonstrated actions either roughly, using the same body part, or very precisely, matching the movement trajectory (*imitation*, e.g. Heyes 2021; Huber et al. 2009). Alternatively, animals have been found to reproduce the result or effect of a demonstration by applying an action other than that used by the model. They thereby learn about certain aspects that are related to the actions of the demonstrator. These aspects may include the action-associated locations or stimuli (*local or stimulus enhancement*, e.g. Avarguès-Weber and Chittka 2014), the properties or potential uses of a stimulus (the so-called ‘affordances’, Gibson 1979; *emulation learning*, Tomasello 1990; later also called *affordance learning*, e.g. Huber et al. 2001; Klein and Zentall 2003), the action’s results or goals (*end-state emulation*, e.g. Auersperg et al. 2014; Tennie et al. 2010), or the movements of objects (*object movement re-enactment*, e.g. Custance et al. 1999; Hopper et al. 2013; social learning mechanisms reviewed in, e.g. Hoppitt and Laland 2008; Whiten 2021; Zentall 2011). While imitative learning mechanisms can only be brought about by the observation of a social agents’ actions, observers may also learn from, or about, changes in the environment rather than actions of the demonstrator through emulative learning mechanisms (end-state emulation, affordance learning, or object movement re-enactment), which may result in observers using their own instead of demonstrated techniques (product-oriented copying; Tennie et al. 2009).

In fact, there might be an important distinction to be made within these emulative learning mechanisms, in terms of (a) from or (b) about what animals learn. While social learning experiments generally test what an observer can learn from a social demonstration *about* actions, *about* affordances, *about* outcomes, or *about* object movements, it is rarely asked whether the social model is even relevant. Especially when learning about objects, observers may learn *from* the changes in the environment, even though a social model is technically the cause of those changes. For example, in a study on domestic pigs (*Sus scrofa domestica*) using a bidirectional control procedure, the observers used different actions and locations for their attempts to open the (blocked) sliding door to the demonstrated direction (Veit et al. 2017). We therefore explained their successful openings of the unblocked sliding door by assuming object movement re-enactment (Custance et al. 1999). While the observers might have learned *about* the movements of the sliding door, it remains unclear whether they required to learn it *from* an acting social agent.

An explicit test for this asocial observational learning is the so-called ghost control test, which features an automagical demonstration of self-moving objects without a social agent present (Heyes et al. 1994; Hopper 2010). However, as social animals, pigs may benefit from seeing a conspecific (*social facilitation*, Thorpe 1957). This can be tested with a somewhat intermediate procedure (socially enhanced ghost control) that involves an inactive social partner who is positioned next to the self-moving objects (e.g. Akins et al. 2002; Fawcett et al. 2002; Klein and Zentall 2003).

So far, domestic pigs have been shown to learn from conspecifics (peers and older generations alike), mainly using local and stimulus enhancement (Nicol and Pope 1994; Oostindjer et al. 2011). Even though pigs are known to be highly interested to manipulate or interact with objects, i.e. through play (Beaudoin et al. 2019; Newberry et al. 1988; Yang et al. 2018), most social learning experiments focussed on piglets learning about food location, flavour, or the mere food intake (e.g. Figueroa et al. 2013, 2020, 2021; Morgan et al. 2001, 2003; Oostindjer et al. 2011), with only two studies having investigated learning about object manipulation (Nicol and Pope 1994; Veit et al. 2017). Similar to many young animals (Van Schaik 2010), piglets might be more prone to learn from conspecific demonstrations compared to adult pigs, who might be more restricted in their ability or willingness to learn from others, as was, for example, found in horses (Krueger et al. 2013). One such restriction poses the social structure in which pigs organize their group life, with an integral part being the dominance hierarchy and social relationships (Goumon et al. 2020; Mcbride et al. 1964). While dominant demonstrators seem to elicit stronger attention, their presence during test situations seems to rather inhibit learned behaviour (Luna et al. 2021b, but see 2021a). In comparison to conspecifics who may be seen as competitors for resources, adult pigs might rather benefit from a social demonstration of their human caregivers.

As a domesticated species, pigs have been selectively bred by humans over thousands of years (Vigne 2011), thereby not only establishing humans as an important source for food and shelter, but also likely leading to an increased tameness towards humans (Collarini et al. 2022). They therefore might be especially attuned to attending to humans. Similar to other domestic animals, like dogs (Miklósi and Soproni 2006), cats (Mäses and Wascher 2022), goats (Kaminski et al. 2005; Nawroth et al. 2020) and horses (Krueger et al. 2011; Proops and McComb 2010), who have already been documented to learn from humans (dogs: e.g. Huber et al. 2009; Miller et al. 2009; cats: Fugazza et al. 2021; goats: Nawroth et al. 2016a; horses: Bernauer et al. 2020; Schuetz et al. 2017; but see Rørvang et al. 2020; Burla et al. 2018), pigs have been found to pay attention to humans and can learn to follow human-given cues like pointing gestures (Bensoussan et al. 2016; Nawroth et al. 2013, 2014; Nawroth et al. 2016a, b; but see Albiach-Serrano et al. 2012; Gerencsér et al. 2019), or object handling (shaking of a food container, Albiach-Serrano et al. 2012), although with inconsistent results due to the large variation in the given cue types (distal/proximal, sustained/momentary), or the subjects’ ages and pre-experiences. Although learning from humans about object manipulation might be additionally difficult due to differing morphologies, pigs might be a likely candidate for possessing heterospecific learning capabilities; especially if social tolerance is a driving factor in observational learning in adult pigs.

In the present study, we investigated how well adult free-ranging domestic pigs learn from varying degrees of social and non-social demonstrations to solve a two-step manipulative foraging task. To solve the task, pigs had to learn to remove a wooden plug from its recess (step 1), to then be able to move a lid covering the top of a food compartment (step 2) where food was hidden. Different groups of observers were either presented with a social demonstration (conspecific or human), a non-social ghost demonstration, or a socially enhanced ghost demonstration. These four observer groups were additionally compared to a non-observer control group as baseline. We predicted that the non-observers would predominantly fail to solve the two-step task. In contrast, observers were expected to learn most, and therefore perform best, when allowed to watch a conspecific demonstrator, due to the advantage of being able to precisely copy the demonstrated actions. To enable observers to act without rank-biassed impairment, demonstrators were moved to a visually occluded compartment during test phases. Observers of the human demonstrator were also expected to learn, albeit with lower fidelity and success due to the differing body parts used (hand instead of snout). In comparison, we predicted that if pigs need social demonstrations, the pigs observing the ghost control groups would perform worse, but still better in social than the non-social condition. We did expect pigs of these two groups to perform better than the non-observers, because they might learn from the moving parts about their affordances and try to re-enact them, as was suggested in Veit et al. (2017).

## Methods

### Animals and housing

We tested 40 (19 male, 21 female) free-ranging domestic pigs (*Sus scofra domestica*) of the Kune Kune breed, known for their small but round stature and very human-friendly demeanour. The pigs were kept in semi-natural conditions in one life-long kin-based group at the Haidlhof Research Station, Bad Vöslau, Lower Austria. Pigs had daily close contact to human caregivers and were trained and handled with positive reinforcement only. The group consisted of three mother sows (born in 2013) and their offspring (born on site in 2014 and 2015). At the time of testing, all subjects had been 5 years or older. Males were vasectomised at the age of five months to prevent inbreeding and maintain natural behaviour regarding hormone levels. Within a 1-hectare forest, the pigs found shelter in six insulated wooden A-shaped huts and could use a wallow for skincare and cooling down. They were fed a daily portion of vegetables and boiled corn and were otherwise free to graze on the clover-grass mixture on an 8-hectare pasture. Well-water was provided at two places in the forest and one place at the pasture ad libitum.

### Apparatus

**Fig. 1:**
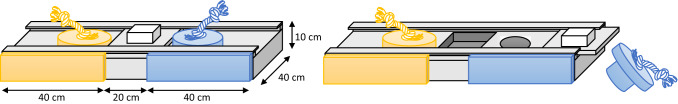
Schematic drawing of the apparatus in closed (left) and open (right) position, as used in part II, with yellow on the left of the apparatus and blue on the right (color figure online)

The apparatus (Fig. 1) was composed of a wooden frame (100 cm × 40 cm × 10 cm) which enclosed a food compartment (20 cm × 20 cm) in the centre. A wooden slider could be moved on top of the food compartment to block access to the reward. For easier manipulation, the slider had a white handle mounted on top (5 cm × 5 cm × 3 cm). The slider was held in place by two rails on the front and backside, and could be blocked from moving left or right by two wooden plugs (Ø 15 cm, 10 cm high) that interchangeably fit into two round recesses. A thick rope of 10 cm length was attached to each of the plugs. The ropes were classic dog toys with two strong knots at both ends. One of the knots was used to attach the rope to the plug. To gain access to the reward, at least one plug needed to be removed by pulling on the rope, before the slider could be pushed to the respective side. To prevent pigs from moving the whole apparatus during the trials, it was fixed to a sturdy wooden board (1.25 m × 1.25 m).

For part II of the experiment, the two plugs were made visibly distinguishable with different colouring (blue and yellow), for the identification of possible enhancement effects (local or stimulus). Furthermore, to increase the coloured surface, two (removable) coloured wooden covers (40 cm × 10 cm) were attached onto the front of the apparatus, facing the observer.

### Procedure—part I

To test their problem-solving ability through trial-and-error learning, 22 pigs (10 male, 12 female) were tested in late 2016 whether they could solve the manipulation task in two steps. Each pig was first presented with a single-step task and, once they had solved it, a two-step task. To gain a reward, pigs first needed to learn through trial and error to slide the horizontal board covering the food compartment to either the left or right side (single step task). Once pigs had solved the single step task, they were in the next trial presented with the same apparatus that now required a two-step manipulation. To be able to move the cover away from the food hole, one of the two wooden plugs blocking the cover had to be removed first by pulling on the attached rope. Trials lasted until the pigs had stopped to interact with the apparatus for more than one minute, after which they were called back into a waiting compartment. Before starting another trial, the pigs got a motivation trial in which the open apparatus was presented, and the reward was free to take. In total, one session of five trials was executed per pig. These tests served as the control condition (non-observer) for the following social learning experiment (part II). Tests were conducted in a square 7.4 m × 7.4 m outdoor arena, bounded by Patura Steckfix hurdles (1.83 m × 0.92 m, ^©^PATURA KG). The outer boundary of the arena and the ground was covered with a dark green plastic tarpaulin. The apparatus was positioned in the middle of the arena.

### Procedure—part II

Four years later, in late 2020, we conducted the second part of this experiment, using the same apparatus, but providing demonstrations prior to testing. The 36 subjects (18 male, 18 female) used in this part had either participated in 2016 (n_pre-experienced_ = 18) but were naïve to the solution as they never were successful in opening the apparatus, or they were completely naïve to the apparatus (n_naïve_ = 18). Subjects were assigned to one of four groups of nine pigs each, accounting for pre-experience, sex and kinship. In the four groups, different types of demonstrations provided varying degrees of social and non-social information (Table 1). Observers were either presented with demonstrations of trained conspecifics, humans, an unseen force (non-social ghost control), or an unseen force accompanied by an inactive conspecific bystander (social ghost control). Each observer of a social demonstration (conspecific, human, social ghost) was presented with their own assigned demonstrator. This was done to enable testing whether pigs can learn from conspecifics or humans on group level, and not only from one specific individual. Human demonstrators were recruited from the pool of people interacting with the pigs on a regular basis (researchers and animal caretakers). Pigs were trained as demonstrator or bystander once they had finished the experiment as a test subject (either part I or part II). For a description of the demonstrator training, see supplementary information.

**Table 1:** Composition of observer groups of experimental part II

Sex	Conspecific		Human		Ghost		Social Ghost		Part I only
Female	^14^Bibi	(YL)	^15^Zirbe	(YL)	^14^Zoe	(YL)	^15^Blossom*	(YL)	^13^Beauty
	^15^Radieschen*	(BL)	^14^Bessy	(BL)	^14^Bijou*	(BL)	^15^Rosine	(BL)	^15^Raya
	^13^Zora*	(YR)	^14^Bella*	(YR)	^14^Rapunzel	(YR)	^14^Zwetschge	(YR)	^13^Rosalie
	^15^Belana	(BR)	^15^Rubina	(YR)	^15^Zita*	(BR)	^15^Bernadette	(BR)	
					^14^Blume*	(YL)	^14^Zafira*	(BL)	
Male	^15^Zoltan	(YL)	^14^Romeo	(YL)	^14^Rasputin*	(YL)	^15^Baldur	(YL)	^14^Zampano
	^15^Zeppelin*	(BL)	^14^Zazou*	(BL)	^15^Zardoz	(BL)	^15^Bolero	(BL)	
	^14^Zacharias*	(YR)	^14^Benjamin*	(YR)	^15^Bruno*	(YR)	^14^Rudi*	(YR)	
	^15^Barbarossa	(BR)	^14^Zerberus*	(BR)	^15^Ronon	(BR)	^15^Zeus*	(BR)	
	^15^Radomir	(BR)	^15^Zafran	(YL)					

Upper half of subjects are female, lower half of subjects are male. Year of birth is indicated in front of the names (2013, 2014, 2015). Kinship is indicated by the first letter of each name (B, R and Z litter). Abbreviations in brackets indicate the colour and side of the removed plug during demonstration: YL, yellow left; BL, blue left; YR, yellow right; BR, blue right. Asterisks mark subjects with pre-experience of the apparatus due to participation in part I. Last column represents the four subjects that did not participate in the second part

In addition to the four demonstration types (conspecific, human, social and non-social ghost), there were four kinds of demonstrations possible with regard to the colour and side of the removed plug, i.e. blue-right, blue-left, yellow-right, or yellow-left. These demonstration combinations of plug colour and side were also roughly the same within the two sexes of each of the four observer groups (see Table 1). The coloured plugs and covers were used to identify possible enhancement effects on either the side (location) of the demonstration or the associated colour (stimulus). To distinguish between local or stimulus enhancement, plugs were switched between demonstrations and tests. This led to a dissociation between the observed stimulus and location. Therefore, observers could only follow the colour cue (stimulus) or the side cue (location). Out of the four demonstration combinations (colour and side of the removed plug during the demonstration), two test configurations (colour and side of both plugs during the test) were possible. Both yellow-left and blue-right demonstration combinations would result in the test configuration blue-left/yellow-right, whereas the demonstration combinations blue-left and yellow-right would result in the test configuration yellow-left/blue-right.

**Fig. 2:**
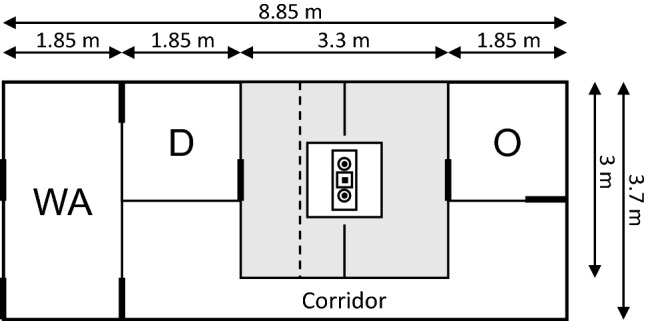
Schematic drawing of the test arena, comprised of a waiting area (WA), demonstrator compartment (D), observer compartment (O), test compartment (grey), and a corridor. Thick lines mark the locations of human-operated doors. The dashed line indicates the position of the stranded wire cables used during the social ghost control demonstration

Tests of experimental part II were conducted in a 3.7 m × 8.85 m outdoor test arena (Fig. 2) made of Patura Steckfix hurdles (1.83 m × 0.92 m, ^©^PATURA KG). The outer boundary of the arena and the ground was covered with a dark green plastic tarpaulin. Pigs entered the test arena from the pasture through the waiting area. Observer pigs were led through a corridor to the 1.85 m × 1.85 m observer compartment, from which they could observe the demonstration and enter the test compartment (3.3 m × 3 m). On the opposite side of the test compartment, another 1.85 m × 1.85 m compartment was located, from which the demonstrator could enter. The apparatus was positioned in the centre of the test compartment between two barriers (0.9 m × 0.6 m) that were installed to deter demonstrator and observer pigs alike from crossing over to the other section.

In total, one session of five trials was conducted per pig. One trial consisted of two phases—the demonstration phase and the test phase. During the demonstration phase, one of the plugs was pulled out, and the cover was slid to the respective side to lay open the food compartment. If the observer had paid attention to the demonstration by directing its head towards the apparatus during plug removal and slider opening, the test phase started, otherwise another demonstration was executed. The number of demonstrations depended on the observer's attention but did not exceed five per demonstration phase. Once the observer had watched a full demonstration, the demonstrator was positioned in the visually separated waiting area to not distract the test subject, and the apparatus was prepared for the test phase. To avoid the experimenter giving additional cues to the observer, the experimenter first pulled a curtain in front of the observer compartment to block the observer's view into the test arena. Then the apparatus was prepared by refilling the food compartment in the centre of the apparatus, sliding the slider on top, and repositioning the plugs and coloured covers in the opposite locations (stimulus transposition). Finally, the curtain was removed, and the observer was released into the test arena to interact with the apparatus for a maximum of five minutes. If interactions had stopped for more than one minute, the observer was called back into the observer compartment to start another trial. Before the next demonstration phase began, the curtain was again pulled in front of the observer, so the experimenter could reset the apparatus to its demonstration composition. The demonstration phase started anew once the observer looked towards the apparatus again.

#### Conspecific and human demonstration

**Fig. 3:**
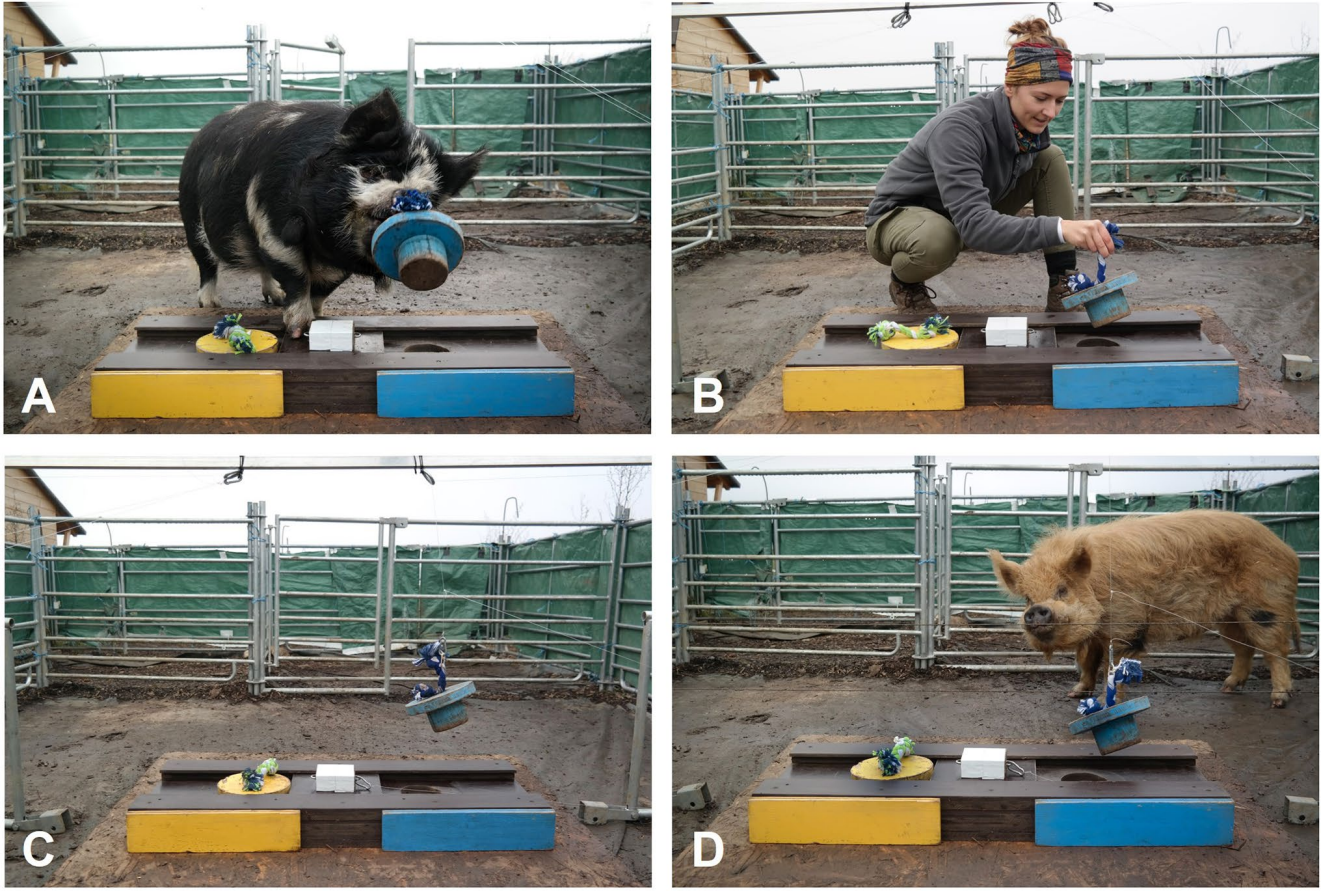
Pictures of the four demonstration types during plug removal. **A**: conspecific demonstration, **B**: human demonstration, **C**: ghost control demonstration, **D**: social ghost control demonstration

Once the test subject was directing its head towards the apparatus, the demonstrator was allowed to enter the test compartment. The demonstrators were trained (conspecific)/instructed (human) to remove the assigned plug by biting into the rope (conspecific)/pulling on the rope with one hand (human) and putting it next to the apparatus (Fig. 3A and B). Demonstrators then had to open the slider with their nose (conspecific)/the same hand used before to remove the plug (human) to push the slider into the removed plug’s direction. The conspecific demonstrator was allowed to eat the food reward, while the human demonstrator took it into their hand and showed it to the observer. The demonstrator was then led to the visually separated waiting area to prevent observers from being affected by their presence during the test phase. If another demonstration was needed due to a lack of attention, the demonstrator would be kept in the demonstrator compartment during resetting the apparatus.

#### Ghost control demonstration

Like the conspecific and human demonstration procedure, one plug was pulled out, and the slider was moved to the respective side. However, no apparent demonstrator was present. To achieve this, the plug and slider were attached to three separate transparent fishing strings that led to the outside of the arena, out of the observer's view. Two fishing strings were attached to the plug’s rope via small metal carabiners, to perform first an upward movement, then a sideways movement. A third string was attached to a small hook at the respective side of the slider handle (Fig. 3C). A hidden person was positioned next to the test arena on the respective side and pulled the strings to open the apparatus. This person stayed hidden during the whole test. Through a small gap between the top rod of the metal fence and the top end of the tarpaulin, the hidden experimenter could see whether the pig was directing their head towards the apparatus. Once this was the case, the experimenter pulled on the respective strings. The fishing strings were removed before each test phase and attached before each demonstration phase.

#### Social ghost control demonstration

As in the ghost control demonstration, the fishing strings enabled a seemingly self-moving apparatus. Additionally, a conspecific bystander was present during the demonstration, who had been selected based on the lack of motivation to interact with the apparatus (Fig. 3D). To furthermore deter the conspecific bystander from interacting with the apparatus during the ghost control demonstration, two barely visible stranded wire cables were spun horizontally across the test arena (see Fig. 2 for location). The wire cables were removed for the test phase.

### Data collection

Two video cameras (JVC Everio GZ-EX515BE) positioned at opposite sides of the test arenas recorded the test subjects’ interactions with the apparatus. Videos of one test session were combined with Reaper (Version 6.25, © Cockos Inc.), and then coded using Solomon Coder (© András Peter, www.solomon.andraspeter.com). All 58 videos (290 trials) were coded by one experimenter, with two further experimenters additionally coding a sample of 17 videos (85 trials, 29.3%). Inter-rater reliability (intraclass correlation coefficient ICC) was good with on average 89.1% (lwrCI = 85.0%, uprCI = 92.3%) agreement (see supplementary information: Table S2).

From the videos we measured the following response variables: latency to observe a full demonstration (it may encompass more than one demonstration), first manipulation side and colour (of first nose contact with the apparatus), the durations of slider and plug manipulation, as well as the durations of plug manipulation techniques “levering” and “pulling”, plug removal (of at least one plug as a measure of success in solving the first step), success (solving the two-step task and getting the food), as well as the latencies to plug removal and success.

### Data analysis

Data were analysed and plotted using R (Version 4.2.2. © 2020 The R Foundation for Statistical Computing). Inter-rater reliability (intraclass correlation coefficient, ICC) was calculated using the R function icc {irr}. To investigate the differences in latencies we used Cox mixed-effects models (R function coxme {coxme}). We considered the latencies and whether plug removal or full success occured or not (using total trial duration if it did not occur). For the variables measuring durations (of slider, plug, levering, and pulling manipulations), we fitted linear (mixed) models assuming normal distribution (R function lmer {lme4}). The variables were log-transformed, before fitting models, to improve fit for linear models assumptions. For the variables measuring events (first interaction side, plug removal, and success), we fitted generalized linear (mixed) models with a binomial error structure and a logit link function (R function glmer {lme4}). Models were fitted for data of the first trial, and the whole session (trials 1–5). Trials of observers in which no interaction with the apparatus took place were excluded from data analysis (not applicable to first trial data, and to the analysis of latency to observe a full demonstration). To account for repeated observations of the same individual across trials, and to avoid pseudo-replication, we included the random intercept effects of individual. To avoid overconfident models and to keep the Type I error rate at the nominal level of 0.05 (Barr et al. 2013), we included the random slope of trial number and its interaction with the random intercept of individual. The interaction between these random slopes and intercepts was removed from the model when they were in part unidentifiable, with absolute correlation parameters estimated as 1 (Matuschek et al. 2017).

We first analysed the effect of the fixed effects of interest demonstration type (conspecific, human, ghost, social ghost), or demonstration colour (blue, yellow) and demonstration side (left, right), on the performance of the observer pigs. Demonstration colour and side were entered into the models as a two-way interaction and were fixed effects of interest only in models on the first interaction side, otherwise they were used as control variables. We furthermore added as a control variable whether a subject had pre-experience with the apparatus due to participation in part I. Additionally, in models on first interaction side we added the first interaction colour to adjust for a possible colour bias, and in models on slider and plug manipulation durations we adjusted for the total duration of manipulating the apparatus. In models on plug levering or plug pulling durations we analysed the effect of having either observed a social agent interacting with the apparatus (conspecific, or human), a ghost demonstration (ghost, or social ghost), or not having observed any demonstration (non-observer). We also adjusted for the total duration of plug manipulations in these models. The covariates trial number, duration of plug manipulations and total manipulation duration were z-transformed before including them into the model to ease model interpretation and model convergence (Schielzeth 2010). In general, the models fitted for the first trial data were reduced in terms of their fixed effects. Due to a lower number of observations, we aimed to reduce model complexity by removing control variables from the models. Covariates remained in the models on first trial data nevertheless.

As an overall test of the effect of the fixed effects of interest, we compared the full model with a null model, using a likelihood ratio test (Dobson 2002). The null model included the contol variables and the same random effects structure as the full model, but lacked the fixed effects of interest (Forstmeier & Schielzeth 2011). Alternatively, if a model only included one fixed effect, tests were derived using a likelihood ratio test (Barr et al. 2013; R function drop1 {stats} with argument ’test’ set to ”Chisq”). Whenever tests revealed a significant effect of the main fixed effects, pairwise comparisons were conducted using the R function emmeans {emmeans}.

We then analysed differences between observer and non-observer data (experimental parts I and II) for all of the measured variables, except first interaction side, again for first trial data and the whole session of five trials. Observer data were pooled to compare to the non-observer data whenever the observer data model revealed no significant differences. We included subject ID as random effect with trial as random slope for analysis of whole session data, and without a random slope for first trial data. The models analysing durations included the respective covariates, which were used in the corresponding observer data models.

For a detailed overview of all fitted models and results see supplementary information (Tables S1–18).

## Results

### Latency to observe a full demonstration

**Fig. 4:**
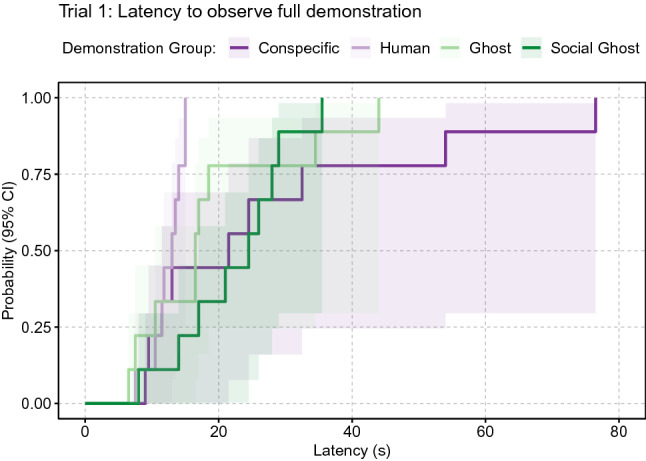
Survival curves of the latency to observe a full demonstration per demonstration type in trial 1

We found a significant effect of demonstration type on the accumulated time to observe a full demonstration during the first trial (full-null model comparison: χ^2^_3_ = 9.413, *P* = 0.024, Table S4a). Here, observers of human demonstrations needed the least amount of time to observe a full demonstration, with a significant difference to observers of the conspecific and social ghost demonstration types (pairwise comparisons: human—conspecific: estimate ± SE = 1.569 ± 0.59, z = 2.661, *P* = 0.039; human—social ghost: estimate ± SE = 1.785 ± 0.65, z = 2.739, *P* = 0.031; Table S4; Fig. 4). Over all five trials, the overall effect of the demonstration type was reduced to a trend (full-null model comparison: χ^2^_3_ = 6.661, *P* = 0.084, Table S3a).

### First interaction location

**Fig. 5:**
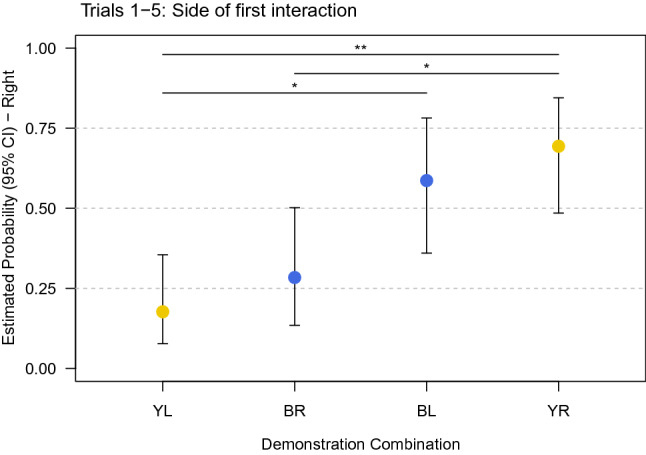
Estimated probabilities to interact first with the right side of the apparatus over five trials per demonstration combination of colour (B—blue, Y—yellow) and side (L—left, R—right). Interactions on the right side are more likely above the 50% level (color figure online)

The location of the first interaction with the apparatus is a proxy for measuring enhancement effects. Analysis of the first manipulation with the apparatus over five trials revealed a significant effect of the interaction between demonstration colour and side of the removed plug (full-null model comparison: χ^2^_3_ = 15.931, *P* = 0.001, model estimate ± SE = 3.627 ± 0.943; Table S5). Only a trend was found for the interaction between demonstration colour and side in the analysis of trial 1 data (full-null model comparison: χ^2^_3_ = 6.23, *P* = 0.098, model estimate ± SE = 4.096 ± 1.917; Table S6). Over all trials, the results show that observers of yellow-left and blue-right plug removal demonstrations (both with the same test configuration: blue-left/yellow-right) preferred to first interact with the left side of the apparatus, whereas observers of blue-left and yellow-right plug removal demonstrations (both with the test configuration yellow-left/blue-right) preferred to choose the right side (Table S5c, Fig. 5). Overall, the preferred location of the first manipulation corresponded to the location of the blue plug during the test phase, not the demonstration phase.

### Manipulation durations

**Fig. 6:**
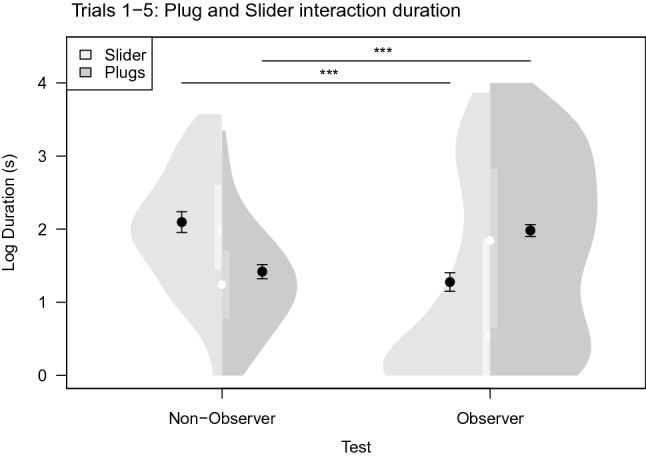
Violin plot of the log duration to interact with plug or slider, relative to the total interaction duration, for non-observer (part I) and observer (part II) over five trials. Violin shapes (grey) indicate the number of cases per log duration. Integrated box plots (lighter grey) show the interquartile range, with the white dots indicating the median. Black dots represent model estimates with 95% CI

No significant effect of demonstration types on plug or slider manipulation durations were found for either first trial data or data over all trials (Tables S7a–S10a). When compared to the observers overall, we found that non-observers interacted longer with the slider, the object under which the reward was located, both in trial 1 (full-null model comparison: χ^2^_1_ = 38.811, *P* < 0.001, model estimate ± SE = − 1.232 ± 0.152; Tables S8d, e) and over all five trials (full-null model comparison: χ^2^_1_ = 112.47, *P* < 0.001, model estimate ± SE = − 0.82 ± 0.068; Tables S7c, d; Fig. 6). Conversely, we found that observers spent more time interacting with the plugs, the objects that needed to be removed first to gain access to the reward, compared to the non-observers, both in trial 1 (full-null model comparison: χ^2^_1_ = 30.834, *P* < 0.001, model estimate ± SE = 0.774 ± 0.12; Tables S10c, d) and over all five trials (full-null model comparison: χ^2^_1_ = 80.506, *P* < 0.001, model estimate ± SE = 0.563 ± 0.058; Tables S9c, d; Fig. 6).

We furthermore found that observers used various techniques to manipulate the plugs, of which two could result in the removal of the plug from the recess. Those two techniques were either the “grab and pull” technique, which had been performed by the conspecific and human demonstrators, or a levering technique in which the pigs would wedge their nose under the rim of the plug to lever it out. In a post hoc data analysis, we compared the usage of pulling and levering techniques between the observers of the two social demonstrations (conspecific and human), the observers of the two ghost demonstrations (social and non-social), and the non-observers. We found a significant difference between the three groups in the time spent with the “grab and pull” technique in their first trial (full-null model comparison: χ^2^_2_ = 7.259, *P* = 0.027; Table S12a), and over all five trials (full-null model comparison: χ^2^_2_ = 16.86, *P* < 0.001; Table S11a). While pairwise comparisons only revealed trends in analysis of first trial data (Table S12c), we found that over all trials observers of social demonstrations showed significantly longer pulling manipulations compared to observers of ghost demonstrations (estimate ± SE = 0.22 ± 0.076, t_138_ = 2.89, *P* = 0.012; Table S11c), and compared to non-observers (estimate ± SE = 0.263 ± 0.064, t_179_ = 4.118, *P* < 0.001; Table S11c). There was no difference in pulling durations between observers of ghost demonstrations and non-observers (estimate ± SE = 0.043 ± 0.059, t_193_ = 0.733, *P* = 0.744; Table S11c). No significant difference between the three groups was found with regard to the levering durations in their first trial (full-null model comparison: χ^2^_2_ = 5.533, *P* = 0.063; Table S12d), and over all trials (full-null model comparison: χ^2^_2_ = 5.104, *P* = 0.078; Table S11d).

### Plug removal

We found no significant effect of demonstration type on the removal of any plug, both for data of the first trial (full-null model comparison: χ^2^_3_ = 1.369, *P* = 0.713; Table S14a) and over all five trials (full-null model comparison: χ^2^_3_ = 2.442, *P* = 0.486; Table S13a). Observers as one group did however perform better than non-observers in removing a plug in their first trial (full-null model comparison: χ^2^_1_ = 9.352, *P* = 0.002; model estimate ± SE = 2.935 ± 1.648; Tables S14c, d), and over all five trials (full-null model comparison: χ^2^_1_ = 58.852, *P* < 0.001; model estimate ± SE = 5.527 ± 1.247; Tables S13c, d). Furthermore, a survival analysis revealed that observers were significantly faster in removing a plug compared to non-observers (full-null model comparison: χ^2^_1_ = 73.149, *P* < 0.001; model estimate ± SE = 4.078 ± 0.638; Tables S15c, d) but did not differ between demonstration types (full-null model comparison: χ^2^_3_ = 2.627, *P* < 0.453; Table S15a).

### Success

**Fig. 7:**
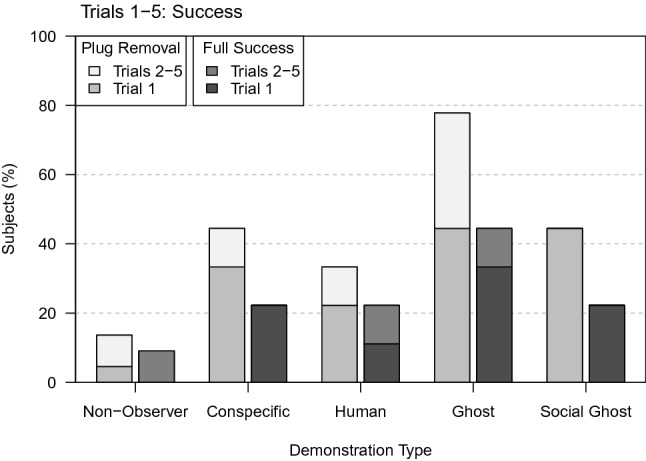
Percentage of test subjects solving step 1 (plug removal) or both steps (full success: plug removal and slider opening) per demonstration type in Trial 1 or later trials

Among the observers, the highest number of successful subjects was in the non-social ghost demonstration type (Fig. 7), whereas the highest number of successful trials was in the group of conspecific observers, with two successful observers solving the task in all five trials. However, we found no effect of demonstration type on the observers’ success to solve the two-step task, both in their first trial (full-null model comparison: χ^2^_3_ = 1.333, *P* = 0.721; Table S17a), and over all trials (full-null model comparison: χ^2^_3_ = 0.144, *P* = 0.986; Table S16a).

Compared to the non-observers, observers were significantly more successful in solving the two-step task over all trials (full-null model comparison: χ^2^_1_ = 64.798, *P* < 0.001; model estimate ± SE = 11.715 ± 3.074; Tables S16c, d). In their first trial, eight of the 36 observers, but none of the 22 non-observers, solved the two-step task (Fig. 7). As none of the non-observers was able to solve the task in the first trial, we could not calculate a GLMM. Instead, the significant difference between the groups was established with a Fisher’s exact test (Fisher’s exact test: *P* = 0.019, N = 58). A survival analysis also revealed that observers were not only more successful than non-observers but were also significantly faster in solving the task (full-null model comparison: χ^2^_1_ = 52.793, *P* < 0.001; Table S18c). Additionally, we found effects of demonstration colour and side, as well as pre-experience on the observers’ latency to solve the task. Observers with pre-experience due to participation in experimental part I, or with demonstrations in which either the blue or the left plug was removed, were faster than their counterparts without experience (coefficient ± SE = 3.32 ± 1.439; Table S18b), or with demonstrations in which either the yellow plug (coefficient ± SE = − 3.132 ± 1.411; Table S18b), or the right plug (coefficient ± SE = − 4.391 ± 1.941; Table S18b) was removed.

## Discussion

In this study, we found that adult domestic pigs could benefit from human, conspecific, and ghost control demonstrations in solving a challenging manipulation task that required two sequential steps. Only with the opportunity to observe a demonstration were pigs able to solve the task in their first trial. While non-observers spent most of their time manipulating the object that was directly blocking the access to the reward (slider), observers diverted their attention and following interaction mostly to the plugs. Compared to eventually successful non-observers, observers were significantly faster in both removing the plug and in completely solving the task. These findings indicate that demonstrations did not only put stronger emphasis on the plugs but provided further information on their relevance and affordances.

While the four observer groups with their respective demonstration types did only marginally differ in their opening success, the techniques used by the pigs to manipulate the plugs differed between groups of social demonstrations (conspecific and human) and ghost demonstrations (social and non-social ghost). Both, non-observers and observers of the two ghost control demonstrations almost exclusively performed the most natural exploration behaviour of pigs—a sort of rooting—whereby they wedged their nose under the rim of the plug to try to remove it. Observers of human and conspecific demonstrators, in turn, performed the demonstrated action of pulling on the rope that was attached to the plug, suggesting some form of social learning (learning about the actions of the demonstrator)—not just social influences like social facilitation or local enhancement—at work. However, their attempts were often not forceful enough, leading them to soon resort to other kinds of manipulations. As success rates ultimately did not differ to the ones by the ghost control groups, we suggest that the pigs learned mostly from the movements of the objects about their affordances (they can be moved), or even their movement trajectory (upwards/sidewards), as this is the common feature of all demonstrations. This points towards a mixture of non-social and social elements in this observational learning study with pigs.

We expected conspecifics to provide the strongest informational values. However, the observers of conspecific demonstrators showed a comparably low success rate with only two out of nine subjects opening the box, same as observers of the human and social ghost demonstrations. This overall low success rate for the task may have been caused by inhibitory influences of the conspecific demonstration. Especially observers of demonstrations with a conspecific present (conspecific and social ghost) showed initially little interest in observing the demonstrations, particularly in comparison to human demonstrators, indicating some form of social inhibition describing a diminished performance under social conditions (Zajonc et al. 1969). It is also possible that some observers were distracted by the presence of the conspecific and thereby failed to learn about important aspects of the demonstration (e.g. Lefebvre and Helder 1997). Social dynamics and hierarchy structure can severely impact the observer’s attention towards demonstrations and motivation to act on the observed behaviour. This is especially true for cases of horizontal transmission. While juveniles are often allowed to forage in close proximity to adults, thereby enabling scrounging of food and information about foraging techniques (e.g. Schiel and Huber 2006; Voelkl et al. 2006), adults have generally less opportunities to watch others from nearby, especially if they are of lower rank than the demonstrator. In pigs, dominant demonstrators seem to elicit stronger attention, but their presence during test situations rather inhibits the learned behaviour (Luna et al. 2021b, but see 2021a). In the present study, we tried to prevent this negative effect by always removing the demonstrator from the visual field of the observer during the test. However, the presence of the demonstrator during the demonstration phase might have exerted enough inhibitory effect on the willingness to observe all relevant aspects of the presented actions. This might have left some observers with only few parts of the information.

Contrary to observers of conspecific demonstrators (conspecific, social ghost), observers of human demonstrators paid very close attention to the demonstration, especially in their first trial. This increased attention might be because the pigs in this study view humans as provider of resources, opposed to conspecifics who may be rather seen as competitors for resources. Not only the general factor of the domestication history, but also the particular living conditions of our subjects have very likely contributed to this result. All pigs had human contact on a daily basis for their entire lives (of five or more years), with strong bonds established to their human caretakers. These strong bonds were forged due to daily social interactions which were often detached from any feeding situation. While a pure hand-food connection might be enough for an increased attention towards humans compared to conspecifics, these bonds might have increased the likelihood of learning relevant aspects of the demonstration, possibly leading more observers to use the “grab and pull” manipulations.

While there was no significant effect of demonstration type on the success latency of the observers, the demonstrated side and colour of the removed plug seemed to have an effect. Results indicated that observers of blue or left plug demonstrations were faster in solving the task compared to observers of yellow or right plug demonstrations. Additionally, we found some indication that the colour blue was a contributing factor for first interaction locations due to its position during the test phase. The higher saliency of the blue colour compared to the yellow certainly contributed to these findings. Previous research on colour discrimination in pigs suggests that blue is the only colour pigs can distinguish from the other two major colours, red and green (Tanida et al. 1991). Furthermore, in an object choice test pigs strongly preferred to interact with a blue ball over a golden ball (Chen et al. 2020). This indicates that blue might pose as a strong attractor in pigs, which might explain why observers performed better when observing blue objects. These colour preferences need to be considered in the design of future tests with pigs.

The saliency of an object due to its colouring might be of particular help to pigs when learning about their environment, as they have been found to have low visual acuity (Zonderland et al. 2008). While Kune Kune pigs in particular might seem an unlikely breed to study visual capabilities, as some of them show morphological peculiarities, i.e. brachycephaly (or airorhynchy in Geiger et al. 2021), which itself may be associated with ocular pathologies (Appelboam 2016), they have been already found to use their vision to learn to distinguish between displayed visual cues using a touch screen apparatus (Wondrak et al. 2018), or to learn from observation of others (Veit et al. 2017). Both of these tests did not require the pigs to see over large distances and presented fairly large stimuli. Similarly, in the present study, demonstrations took place around 1.8 m away from the observers’ head, with objects of 15 cm in diameter. Of course, the effect of ageing in eyesight should not be overlooked, as the previous studies were conducted when the pigs were still young, in contrast to the present study. This might be one contributing factor why the pigs of this study were not as successful. They might have had some difficulties recognizing the exact details of the demonstration, in particular the social one, which might have led some of them to being unable to reproduce the demonstrated behaviour by the conspecific and human demonstrators. Pigs with ghost control demonstrations might have had a clearer view of the objects, which in turn might have resulted in the slightly better performance in at least the non-social ghost observers.

Surprisingly, there seemed to be an effect of pre-experience on the observers’ success latency. Based on the current literature, pigs are known to possess long-term memory of objects (5 days: Gifford et al. 2007) and also manipulation techniques of objects (5 months: Veit et al. 2017). Although our study would require long-term memory of 4 years, there is currently no evidence to support the notion that this is impossible for pigs. If so, it might be that due to the previous interactions with the apparatus, the observers with pre-experience were informed about some aspects of the task which were less apparent to the naïve observers. For example, contrary to naïve observers, pre-experienced pigs had received food rewards from within the open apparatus in between the non-observer test trials. This reward-related memory might have facilitated the affordance learning when presented with the demonstrations on the removal of the plugs. This effect of pre-experience was only represented in the success latency, not the probability to succeed, possibly due to the overall low success rate and other influencing factors.

In conclusion, we provided the first evidence of adult domestic pigs learning how to manipulate objects not only from social (conspecific and heterospecific) demonstrations, but also from ghost control demonstrations, the latter indicating the use of emulative learning (through the object’s movements). Observers likely had learned about the affordances of the plugs that non-observers were not able to infer. In addition to the levering technique that was not demonstrated, but which was preferred by both groups of ghost control observers, observers of human and conspecific demonstrators used the demonstrated “grab and pull” technique, indicating that the demonstrators’ actions also had an influence on the later performance of observers. As test group sample sizes were low, further studies are necessary to provide conclusive evidence on heterospecific social learning in pigs. Overall, in this study social learning effects were modest, possibly due to social inhibition or distraction by the conspecific demonstrators (and bystanders). Pigs seemed to be most successful when observing the non-social ghost demonstration, indicating their observational learning of object-related behaviours likely emerges in rather non-social settings, or with particularly close social partners. The pigs’ attention was captivated most by the human demonstrators. If kept with close positive human contact, pigs may be able to not only use human-given cues but also learn from humans how to interact with certain objects in their often-artificial environment. Pigs might also have benefitted from information gathered in previous interactions with the apparatus, which they had retained for four years, providing additional evidence for long-term memory related to objects in pigs. These findings are rare, as most pigs are not exceeding the life span of 6 months. Future research on pigs should shift the focus onto adult and socially well-educated pigs, to broaden our understanding of their cognitive capabilities.

## Supplementary Information

Below is the link to the electronic supplementary material.

Supplementary file1 (PDF 640 kb)

## Data Availability

The datasets generated during and/or analysed during the current study are available from the corresponding author on reasonable request.
