# Supplementary material for: Emulative learning of a two-step task in free-ranging domestic pigs
Source: Anim Cogn. 2023 Jan 18;26(3):929–42. doi: 10.1007/s10071-022-01740-3 (PMC10066142; doi:10.1007/s10071-022-01740-3)
Supplement: Supplementary file 1 — Supplementary file1 (PDF 640 kb) [file 10071_2022_1740_MOESM1_ESM.pdf]

Supplementary Material

Contents

Demonstrator and bystander training 2
Table S1. Description of data 3
Table S2. Inter-rater reliability test. 3
Table S3 a-b: Latency to observe full demonstration (Trials 1-5, observer) 4
Table S4 a-c: Latency to observe full demonstration (Trial 1, observer) 4
Table S5 a-c: Location of first interaction (Trials 1-5, observer) 5
Table S6 a-b: Location of first interaction (Trial 1, observer) 5
Table S7 a-d: Slider interaction duration (Trials 1-5) 6
Table S8 a-e: Slider interaction duration (Trial 1) 7
Table S9 a-d: Plug manipulation duration (Trials 1-5) 8
Table S10 a-d: Plug manipulation duration (Trial 1) 9
Table S11 a-e: Plug pull or lever duration (Trials 1-5) 10
Table S12 a-e: Plug pull or lever duration (Trial 1) 11
Table S13 a-d: Plug removal (Trials 1-5) 12
Table S14 a-d: Plug removal (Trial 1) 13
Table S15 a-d: Latency to plug removal (Trials 1-5) 14
Table S16 a-d: Success (Trials 1-5) 15
Table S17 a-c: Success (Trial 1) 16
Table S18 a-d: Latency to success (Trials 1-5) 17

Demonstrator and bystander training

To train the conspecific demonstrators to remove their respective plug from the apparatus, the first step was to make the plug interesting for the pigs. For this, we used shaping with "clicker-training". To be rewarded, pigs had to initially touch the plug, gradually working their way towards biting into the rope of the plug and pulling on it. At this stage, the plug was held by the trainer, outside of the apparatus context. After biting and pulling the rope of the plug was learned, the demonstrator was presented with a few apple pieces in the open food compartment of the apparatus (slider and plugs removed). Then, to train the pig to open the slider to the respective side the slider was installed, in addition to the non-assigned (blocked) plug. Once the pig was successful in sliding open the food compartment without interacting with the non-assigned plug, the removable plug with which the pig was trained was installed. The training was finished once the pig had learned the procedure of (1) entering the test arena, (2) pulling the assigned plug out of the recess, (3) opening the slider to the assigned direction, and (4) returning to the demonstrator compartment after eating the reward. Every test subject was assigned its own conspecific demonstrator. As a result, we trained nine pigs to open the apparatus, each with a specific side-colour combination. Which pig became a demonstrator, or a bystander, depended on the performance in the previous test. The selection criteria were a combination of the interest in the box at all over the trials and the success in the test. While pigs were selected to become demonstrators if they were either successful or particularly manipulative, the bystander pigs in contrast were chosen based on their lower motivation to interact with the apparatus. The bystander pigs were trained to stay behind two wires spun across the length of the apparatus and not interact with the apparatus or going near the observer.

Table S1. Description of data

To enable interpretation of model results (Tables S2-18).

Data sets	Observations	Excluded Obs.	Comment
Trial 1	58	0	22 subjects in phase I, 36 in phase II
Trials 1-5	289	56	5 trials per 58 subjects, trial 5 missing for one phase I subject
Trial 1, observer only	36	0	first trial of 36 phase II subjects
Trials 1-5, observer only	180	25	5 trials for 36 phase II subjects
**Dependent variables**	Abbreviation	Data type	Levels
Latency to observe full demonstration	dem.L	continuous	
First interaction side	int1.side	factor	2 (l: left, r: right)
Slider manipulation duration	slider.int.D	continuous	
Plug manipulation duration	plugs.int.D	continuous	
Plug levering duration	plugs.lever.D	continuous	
Plug pulling duration	plugs.bitepull.D	continuous	
Plug removal	plugs.rem	factor	2 (0: no, 1: yes)
Latency to plug removal	plugs.rem.L	continuous	
Success	success	factor	2 (0: no, 1: yes)
Latency to success	success.L	continuous	
**Fixed effects**	Abbreviation	Data type	Levels
Demonstration type	dem.type	factor	4 (c: conspecific, h: human, gc: ghost control, sg: social ghost)
Demonstration side	dem.side	factor	2 (l: left, r: right)
Demonstration colour	dem.col	factor	2 (b: blue, y: yellow)
Test phase	test	factor	2 (non-observer: phase I, observer: phase II)
Test group	dem.sgn	factor	3 (social: human & conspecific, ghost: ghost & social ghost, non-observer)
Pre-experience	pre.exp	factor	2 (0: no, 1: yes), 18 subjects
First interaction colour	int1.col	factor	2 (b: blue, y: yellow)
Total manipulation duration	z.total.int.D	continuous	
Plug manipulation duration	z.plugs.int.D	continuous	
Trial number	z.trial	integer	
**Random effect**	Abbreviation	Data type	Levels
Pig identity	pig.id	factor	36 (observer), 40 (incl. non-observer)

Table S2. Inter-rater reliability test.

Rater: 3, Observations: 85

icc(data, model = "twosided", type = "agreement")

Variable	ICC	lwrCI	uprCI
success	1	1	1
plugs.rem	1	1	1
total.int.D	0.959	0.933	0.974
plugs.intD	0.913	0.870	0.942
slider.intD	0.657	0.552	0.748
int1.col	0.734	0.636	0.814
int1.side	0.755	0.663	0.830
success.L	0.999	0.999	1.000
plugs.rem.L	0.999	0.995	1.000
**Mean**	**0.891**	**0.850**	**0.923**

Table S3 a-b: Latency to observe full demonstration (Trials 1-5, observer)

Coxme Model (censor=1): Number of observations: 180, groups: pig.id, 36
Full Model: dem.L ~ dem.type + dem.col * dem.side + pre.exp + z.trial + (1 + z.trial | pig.id)
Null Model: dem.L ~ dem.col * dem.side + pre.exp + z.trial + (1 + z.trial | pig.id)

Table S3a. Analysis of Deviance Table

Model	logLik	Chisq	Df	Pr(>Chisq)	Sign.
Null	-752.59				
Full	-749.26	6.661	3	0.084	.

Table S3b. Model Coefficients

	Coefficient	SE	z value	Pr(>|z|)	Sign.
dem.typegc	-0.078	0.313	-0.25	0.80	
dem.typeh	0.686	0.321	2.14	0.03	*
dem.typesg	-0.023	0.308	-0.07	0.94	
dem.coly	0.181	0.305	0.59	0.55	
dem.sider	0.060	0.324	0.18	0.85	
pre.exp1	0.182	0.221	0.82	0.41	
z.trial	-0.009	0.080	-0.11	0.91	
dem.coly:dem.sider	-0.497	0.447	-1.11	0.27	

Table S4 a-c: Latency to observe full demonstration (Trial 1, observer)

Coxme Model (censor=1): Number of observations: 36
Full Model: dem.L ~ dem.type + dem.col * dem.side + pre.exp
Null Model: dem.L ~ dem.col * dem.side + pre.exp

Table S4a. Analysis of Deviance Table

Model	logLik	Chisq	Df	Pr(>Chisq)	Sign.
Null	-86.898				
Full	-91.604	9.413	3	0.024	*

Table S4b. Model Coefficients

	Coefficient	SE	z value	Pr(>|z|)	Sign.
dem.typegc	0.463	0.514	0.901	0.368	
dem.typeh	1.570	0.590	2.661	0.008	**
dem.typesg	-0.215	0.563	-0.382	0.702	
dem.coly	0.582	0.540	1.078	0.281	
dem.sider	1.413	0.612	2.309	0.021	*
pre.exp1	-0.690	0.355	-1.943	0.052	.
z.trial	-1.066	0.764	-1.394	0.163	

Table S4c. Pairwise Comparisons

Contrast	Estimate	SE	Df	t value	Pr(>|t|)	Sign.
c - gc	-0.463	0.514	Inf	-0.901	0.804	
c - h	-1.569	0.590	Inf	-2.661	0.039	*
c - sg	0.215	0.563	Inf	0.382	0.981	
gc - h	-1.106	0.576	Inf	-1.920	0.219	
gc - sg	0.679	0.542	Inf	1.251	0.594	
h - sg	1.785	0.652	Inf	2.739	0.031	*

Table S5 a-c: Location of first interaction (Trials 1-5, observer)

GLMM: Number of observations: 155, groups: pig.id, 36
Full Model: int1.side ~ dem.col * dem.side + int1.col + z.trial + (1 + z.trial || pig.id)
Null Model: int1.side ~ int1.col + z.trial + (1 + z.trial || pig.id)

Table S5a. Analysis of Deviance Table

Model	npar	AIC	BIC	logLik	deviance	Chisq	Df	Pr(>Chisq)	Sign.
Null	5	201.82	217.03	-95.908	191.82				
Full	8	191.89	216.23	-87.943	175.89	15.931	3	0.001	**

Table S5b. Model Coefficients

	Estimate	SE	z value	Pr(>|z|)	Sign.
(Intercept)	0.350	0.472	0.742	0.458	
dem.coly	-1.885	0.651	-2.897	0.004	**
dem.sider	-1.274	0.639	-1.996	0.046	*
int1.coly	-0.984	0.434	-2.265	0.024	*
z.trial	0.222	0.193	1.154	0.248	
dem.coly:dem.sider	3.627	0.943	3.848	0.0001	***

Table S5c. Pairwise Comparisons

Contrast	Estimate	SE	Df	z value	Pr(>|z|)	Sign.
b l - y l	1.885	0.651	Inf	2.897	0.020	*
b l - b r	1.274	0.639	Inf	1.996	0.190	
b l - y r	-0.467	0.580	Inf	-0.805	0.852	
y l - b r	-0.611	0.655	Inf	-0.933	0.787	
y l - y r	-2.352	0.646	Inf	-3.64	0.002	**
b r - y r	-1.741	0.636	Inf	-2.736	0.032	*

Table S6 a-b: Location of first interaction (Trial 1, observer)

GLM: Number of observations: 36
Full Model: int1.side ~ dem.col * dem.side + int1.col
Null Model: int1.side ~ int1.col

Table S6a. Analysis of Deviance Table

Model	Df	Resid. Dev	Df	Deviance	Pr(>Chi)	Sign.
Null	31	37.468				
Full	34	43.765	-3	-6.297	0.098	.

Table S6b. Model Coefficients

	Estimate	SE	z value	Pr(>|z|)	Sign.
(Intercept)	0.877	0.846	1.036	0.300	
dem.coly	-2.069	1.137	-1.820	0.069	.
dem.sider	-2.713	1.355	-2.002	0.045	*
int1.coly	-1.421	0.964	-1.474	0.140	
dem.coly:dem.sider	4.096	1.917	2.136	0.033	*

Table S7 a-d: Slider interaction duration (Trials 1-5)

LMM1: Number of observations: 155, groups: pig.id, 36
Full Model: slider.int.D.log ~ dem.type + dem.col * dem.side + pre.exp + z.total.int.D.log + z.trial + (1 + z.total.int.D.log + z.trial || pig.id)
Null Model: slider.int.D.log ~ dem.col * dem.side + pre.exp + z.total.int.D.log + z.trial + (1 + z.total.int.D.log + z.trial || pig.id)

Table S7a. Analysis of Deviance Table

Model	npar	AIC	BIC	logLik	deviance	Chisq	Df	Pr(>Chisq)	Sign.
Null	11	303.16	336.64	-140.58	281.16				
Full	14	307.06	349.67	-139.53	279.06	2.098	3	0.552	

Table S7b. Model Coefficients

	Estimate	SE	Df	t value	Pr(>|t|)	Sign.
(Intercept)	1.241	0.177	32.735	7.002	5.46E-08	***
dem.typegc	-0.038	0.174	34.129	-0.217	0.829	
dem.typeh	-0.232	0.175	33.118	-1.322	0.195	
dem.typesg	-0.118	0.177	36.291	-0.666	0.510	
dem.coly	-0.128	0.172	35.203	-0.744	0.462	
dem.sider	-0.058	0.176	30.122	-0.329	0.745	
pre.exp1	0.225	0.124	31.620	1.815	0.079	.
z.total.int.D.log	0.986	0.070	33.256	14.092	1.40E-15	***
z.trial	0.148	0.048	129.477	3.054	0.003	**
dem.coly:dem.sider	-0.082	0.246	31.396	-0.334	0.741	

LMM2: Number of observations: 264, groups: pig.id, 40
Full Model: slider.int.D.log ~ test + z.total.int.D.log + z.trial + (1 + z.trial + z.total.int.D.log | pig.id)
Null Model: slider.int.D.log ~ z.total.int.D.log + z.trial + (1 + z.trial + z.total.int.D.log | pig.id)

Table S7c. Analysis of Deviance Table

Model	npar	AIC	BIC	logLik	deviance	Chisq	Df	Pr(>Chisq)	Sign.
Null	10	549.28	585.04	-264.64	529.28				
Full	11	438.81	478.15	-208.41	416.81	112.47	1	< 2.2E-16	***

Table S7d. Model Coefficients

	Estimate	SE	Df	t value	Pr(>|t|)	Sign.
(Intercept)	2.097	0.072	53.398	29.22	<2E-16	***
testObserver	-0.820	0.068	222.586	-12.08	<2E-16	***
z.total.int.D.log	0.959	0.058	36.572	16.62	<2E-16	***
z.trial	0.067	0.035	37.514	1.9	0.065	.

Table S8 a-e: Slider interaction duration (Trial 1)

LM: Number of observations: 36
Full Model: slider.int.D.log ~ dem.type + z.total.int.D.log
Null Model: slider.int.D.log ~ z.total.int.D.log

Table S8a. Analysis of Deviance Table

Model	Res.Df	RSS	Df	Sum Sq	Pr(>Chi)	Sign.
Null	31	12.68				
Full	34	16.22	-3	-3.541	0.034	*

Table S8b. Model Coefficients

	Estimate	SE	t value	Pr(>|t|)	Sign.
(Intercept)	1.669	0.215	7.767	9.18E-09	***
dem.typegc	0.231	0.310	0.746	0.461	
dem.typeh	-0.584	0.307	-1.905	0.066	.
dem.typesg	0.122	0.302	0.404	0.689	
z.total.int.D.log	0.779	0.115	6.796	1.30E-07	***

Table S8c. Pairwise Comparisons

Contrast	Estimate	SE	Df	t value	Pr(>|t|)	Sign.
c - sg	-0.122	0.302	31	-0.404	0.977	
gc - h	0.815	0.302	31	2.701	0.052	.
gc - sg	0.109	0.315	31	0.347	0.985	
h - sg	-0.706	0.311	31	-2.274	0.126	

LMM: Number of observations: 58, groups: pig.id, 40
Full Model: slider.int.D.log ~ test + z.total.int.D.log + (1 | pig.id)
Null Model: slider.int.D.log ~ z.total.int.D.log + (1 | pig.id)

Table S8d. Analysis of Deviance Table

Model	npar	AIC	BIC	logLik	deviance	Chisq	Df	Pr(>Chisq)	Sign.
Null	4	153.52	161.76	-72.76	145.52				
Full	5	116.71	127.01	-53.36	106.71	38.811	1	4.67E-10	***

Table S8e. Model Coefficients

	Estimate	SE	Df	t value	Pr(>|t|)	Sign.
(Intercept)	3.005	0.131	57.269	22.877	< 2E-16	***
testObserver	-1.232	0.152	26.749	-8.087	1.17E-08	***
z.total.int.D.log	0.772	0.086	57.288	9.02	1.37E-12	***

Table S9 a-d: Plug manipulation duration (Trials 1-5)

LMM: Number of observations: 155, groups: pig.id, 36

Full Model: plugs.int.D.log ~ dem.type + dem.col * dem.side + pre.exp + z.total.int.D.log + z.trial + (1 + z.total.int.D.log + z.trial || pig.id)

Null Model: plugs.int.D.log ~ dem.col * dem.side + pre.exp + z.total.int.D.log + z.trial + (1 + z.total.int.D.log + z.trial || pig.id)

Table S9a. Analysis of Deviance Table

Model	npar	AIC	BIC	logLik	deviance	Chisq	Df	Pr(>Chisq)	Sign.
Null	11	187.4	220.88	-82.701	165.4				
Full	14	189.57	232.18	-80.785	161.57	3.832	3	0.2801	

Table S9b. Model Coefficients

	Estimate	SE	Df	t value	Pr(>|t|)	Sign.
(Intercept)	1.703	0.108	39.743	15.789	<2E-16	***
dem.typegc	0.190	0.104	38.853	1.823	0.076	.
dem.typeh	0.170	0.105	38.400	1.618	0.114	
dem.typesg	0.098	0.105	39.595	0.935	0.355	
dem.coly	0.090	0.102	38.834	0.875	0.387	
dem.sider	-0.141	0.108	37.800	-1.308	0.199	
pre.exp1	-0.068	0.074	37.129	-0.918	0.364	
z.total.int.D.log	1.178	0.037	137.834	31.425	<2E-16	***
z.trial	-0.008	0.035	147.746	-0.221	0.826	
dem.coly:dem.sider	0.141	0.147	36.023	0.956	0.345	

LMM: Number of observations: 264, groups: pig.id, 40

Full Model: plugs.int.D.log ~ test + z.total.int.D.log + z.trial + (1 + z.total.int.D.log + z.trial || pig.id)

Null Model: plugs.int.D.log ~ z.total.int.D.log + z.trial + (1 + z.total.int.D.log + z.trial || pig.id)

Table S9c. Analysis of Deviance Table

Model	npar	AIC	BIC	logLik	deviance	Chisq	Df	Pr(>Chisq)	Sign.
Null	7	412.83	437.87	-199.42	398.83				
Full	8	334.33	362.93	-159.16	318.33	80.506	1	< 2.2E-16	***

Table S9d. Model Coefficients

	Estimate	SE	Df	t value	Pr(>|t|)	Sign.
(Intercept)	1.419	0.050	86.183	28.651	<2E-16	***
testObserver	0.563	0.058	259.819	9.703	<2E-16	***
z.total.int.D.log	1.072	0.032	230.776	33.704	<2E-16	***
z.trial	0.005	0.029	256.921	0.155	0.877	

Table S10 a-d: Plug manipulation duration (Trial 1)

LM: Number of observations: 36
Full Model: plugs.int.D.log ~ dem.type + total.int.D.log
Null Model: plugs.int.D.log ~ total.int.D.log

Table S10a. Analysis of Deviance Table

Model	Res.Df	RSS	Df	Sum of Sq	Pr(>Chi)	Sign.
Null	34	4.801				
Full	31	3.912	3	0.889	0.071	.

Table S10b. Model Coefficients

	Estimate	SE	t value	Pr(>|t|)	Sign.
(Intercept)	2.781	0.119	23.294	< 2E-16	***
dem.typegc	-0.222	0.172	-1.287	0.208	
dem.typeh	0.174	0.170	1.021	0.315	
dem.typesg	-0.181	0.168	-1.079	0.289	
z.total.int.D.log	0.778	0.064	12.228	2.14E-13	***

LMM: Number of observations: 58, groups: pig.id, 40
Full Model: plugs.int.D.log ~ test + total.int.D.log + (1 | pig.id)
Null Model: plugs.int.D.log ~ total.int.D.log + (1 | pig.id)

Table S10c. Analysis of Deviance Table

Model	npar	AIC	BIC	logLik	deviance	Chisq	Df	Pr(>Chisq)	Sign.
Null	4	105.876	114.118	-48.938	97.876				
Full	5	77.042	87.344	-33.521	67.042	30.834	1	2.81E-08	***

Table S10d. Model Coefficients

	Estimate	SE	Df	t value	Pr(>|t|)	Sign.
(Intercept)	2.108	0.094	57.967	22.398	< 2E-16	***
testObserver	0.774	0.120	40.497	6.423	1.14E-07	***
z.total.int.D.log	0.759	0.059	55.241	12.768	< 2E-16	***

Table S11 a-e: Plug pull or lever duration (Trials 1-5)

LMM: Number of observations: 233, groups: pig.id, 40

Full Model:	plugs.bitepull.D.log ~ dem.sgn +	z.plugs.int.D.log + z.trial + (1 + z.plugs.int.D.log + z.trial || pig.id)
Null Model:	plugs.bitepull.D.log ~	z.plugs.int.D.log + z.trial + (1 + z.plugs.int.D.log + z.trial || pig.id)

Table S11a. Analysis of Deviance Table

Model	npar	AIC	BIC	logLik	deviance	Chisq	Df	Pr(>Chisq)	Sign.
Null	7	236.45	260.6	-111.22	222.45				
Full	9	223.59	254.65	-102.79	205.59	16.86	2	0.0002	***

Table S11b. Model Coefficients

	Estimate	SE	Df	t value	Pr(>|t|)	Sign.
(Intercept)	0.323	0.054	84.524	5.931	6.38E-08	***
dem.sgnghost	-0.220	0.073	134.499	-2.997	0.003	**
dem.sgnno	-0.263	0.062	173.293	-4.253	3.45E-05	***
z.plugs.int.D.log	0.249	0.063	37.019	3.981	3.08E-04	***
z.trial	0.047	0.028	25.277	1.693	0.103	

Table S11c. Pairwise Comparisons

contrast	estimate	SE	Df	t value	Pr(>|t|)	Sign.
social - ghost	0.220	0.076	138	2.890	0.012	*
social - no	0.263	0.064	179	4.118	0.0002	***
ghost - no	0.043	0.059	193	0.733	0.744	

LMM: Number of observations: 233, groups: pig.id, 40

Full Model:	plugs.lever.D.log ~ dem.sgn +	z.plugs.int.D.log + z.trial + (1 + z.plugs.int.D.log + z.trial || pig.id)
Null Model:	plugs.lever.D.log ~	z.plugs.int.D.log + z.trial + (1 + z.plugs.int.D.log + z.trial || pig.id)

Table S11d. Analysis of Deviance Table

Model	npar	AIC	BIC	logLik	deviance	Chisq	Df	Pr(>Chisq)	Sign.
Null	7	440.3	464.45	-213.15	426.3				
Full	9	439.19	470.25	-210.6	421.19	5.104	2	0.078	.

Table S11e. Model Coefficients

	Estimate	SE	Df	t value	Pr(>|t|)	Sign.
(Intercept)	0.735	0.098	67.892	7.519	1.65E-10	***
dem.sgnghost	-0.057	0.128	132.976	-0.446	0.656	
dem.sgnno	-0.218	0.107	193.756	-2.043	0.042	*
z.plugs.int.D.log	0.571	0.060	37.552	9.488	1.62E-11	***
z.trial	0.062	0.039	201.683	1.596	0.112	

Table S12 a-e: Plug pull or lever duration (Trial 1)

LMM: Number of observations: 58, groups: pig.id, 40
Full Model: plugs.bitepull.D.log ~ dem.sgn + z.plugs.int.D.log + (1 | pig.id)
Null Model: plugs.bitepull.D.log ~ z.plugs.int.D.log + (1 | pig.id)

Table S12a. Analysis of Deviance Table

Model	npar	AIC	BIC	logLik	deviance	Chisq	Df	Pr(>Chisq)	Sign.
Null	4	113.17	121.42	-52.588	105.175				
Full	6	109.92	122.28	-48.958	97.917	7.259	2	0.027	*

Table 12b. Model Coefficients

	Estimate	SE	Df	t value	Pr(>|t|)	Sign.
(Intercept)	0.637	0.135	58	4.715	1.56E-05	***
dem.sgnghost	-0.462	0.189	58	-2.439	0.018	*
dem.sgnno	-0.450	0.184	58	-2.438	0.018	*
z.plugs.intD.tot.log	0.332	0.077	58	4.318	6.23E-05	***

Table 12c. Pairwise Comparisons

Contrast	Estimate	SE	Df	t value	Pr(>|t|)	Sign.
social - ghost	0.4621	0.202	62.3	2.283	0.0656	.
social - no	0.4495	0.195	50	2.305	0.0643	.
ghost - no	-0.0126	0.19	47.8	-0.066	0.9976	

LMM: Number of observations: 58, groups: pig.id, 40
Full Model: plugs.lever.D.log ~ dem.sgn + z.plugs.int.D.log + (1 | pig.id)
Null Model: plugs.lever.D.log ~ z.plugs.int.D.log + (1 | pig.id)

Table S12d. Analysis of Deviance Table

Model	npar	AIC	BIC	logLik	deviance	Chisq	Df	Pr(>Chisq)	Sign.
Null	4	138.25	146.49	-65.124	130.25				
Full	6	136.72	149.08	-62.358	124.72	5.533	2	0.063	.

Table 12e. Model Coefficients

	Estimate	SE	Df	t value	Pr(>|t|)	Sign.
(Intercept)	0.548	0.170	58	3.219	0.002	**
dem.sgnghost	0.454	0.239	58	1.9	0.062	.
dem.sgnno	0.528	0.232	58	2.271	0.027	*
z.plugs.intD.tot.log	0.592	0.097	58	6.112	8.92E-08	***

Table S13 a-d: Plug removal (Trials 1-5)

GLMM: Number of observations: 155, groups: pig.id, 36

Full Model: plug.rem ~ dem.type + dem.col * dem.side + pre.exp + z.trial + (1 + z.trial || pig.id)

Null Model: plug.rem ~ dem.col * dem.side + pre.exp + z.trial + (1 + z.trial || pig.id)

Table S13a. Analysis of Deviance Table

Model	npar	AIC	BIC	logLik	deviance	Chisq	Df	Pr(>Chisq)	Sign.
Null	8	156.53	180.88	-70.27	140.53				
Full	11	160.09	193.57	-69.05	138.09	2.442	3	0.486	

Table S13b. Model Coefficients

	Estimate	SE	z value	Pr(>|z|)	Sign.
(Intercept)	-2.136	1.905	-1.121	0.262	
dem.typegc	1.310	1.782	0.735	0.462	
dem.typeh	-1.409	1.891	-0.745	0.456	
dem.typesg	-0.337	1.813	-0.186	0.853	
dem.coly	-2.139	1.815	-1.179	0.239	
dem.sider	-0.558	1.786	-0.313	0.755	
pre.exp1	1.957	1.338	1.463	0.144	
z.trial	-0.251	0.276	-0.908	0.364	
dem.coly:dem.sider	1.792	2.582	0.694	0.488	

GLMM: Number of observations: 264, groups: pig.id, 40

Full Model: plug.rem ~ test + z.trial + (1 + z.trial || pig.id)

Null Model: plug.rem ~ z.trial + (1 + z.trial || pig.id)

Table S13c. Analysis of Deviance Table

Model	npar	AIC	BIC	logLik	deviance	Chisq	Df	Pr(>Chisq)	Sign.
Null	4	248.04	262.34	-120.02	240.04				
Full	5	191.19	209.07	-90.59	181.19	58.852	1	1.70E-14	***

Table S13d. Model Coefficients

	Estimate	SE	z value	Pr(>|z|)	Sign.
(Intercept)	-7.486	1.636	-4.576	4.73E-06	***
testObserver	5.527	1.247	4.432	9.34E-06	***
z.trial	-0.219	0.244	-0.898	0.369	

Table S14 a-d: Plug removal (Trial 1)

GLM: Number of observations: 36
Full Model: plug.rem ~ dem.type

Table S14a. Single term deletions

	AIC	Deviance	Df	LRT	Pr(>Chi)	Sign.
<none>	53.723	45.723				
dem.type	49.092	47.092	3	1.369	0.713	

Table S14b. Model Coefficients

	Estimate	SE	z value	Pr(>|z|)	Sign.
(Intercept)	-0.693	0.707	-0.980	0.327	
dem.typegc	0.470	0.975	0.482	0.630	
dem.typeh	-0.560	1.069	-0.523	0.601	
dem.typesg	0.470	0.975	0.482	0.630	

GLMM: Number of observations: 58, groups: pig.id, 40
Full Model: plug.rem ~ test + (1 | pig.id)
Null Model: plug.rem ~ (1 | pig.id)

Table S14c. Analysis of Deviance Table

Model	npar	AIC	BIC	logLik	deviance	Chisq	Df	Pr(>Chisq)	Sign.
Null	2	68.109	72.23	-32.055	64.109				
Full	3	60.758	66.939	-27.379	54.758	9.352	1	0.002	**

Table S14d. Model Coefficients

	Estimate	SE	z value	Pr(>|z|)	Sign.
(Intercept)	-3.71	2.015	-1.841	0.0656	.
testObserver	2.935	1.648	1.781	0.0749	.

Table S15 a-d: Latency to plug removal (Trials 1-5)

Coxme Model (censor=plug.rem): Number of observations: 155, groups: pig.id, 36
Full Model: plug.rem.L ~ dem.type + dem.col * dem.side + pre.exp + z.trial + (1 + z.trial | pig.id)
Null Model: plug.rem.L ~ dem.col * dem.side + pre.exp + z.trial + (1 + z.trial | pig.id)

Table S15a. Analysis of Deviance Table

Model	loglik	Chisq	Df	P(>|Chi|)	Sign.
Null	-207.77				
Full	-206.46	2.6265	3	0.453	

Table S15b. Model Coefficients

	Coefficient	SE	z value	Pr(>|z|)	Sign.
dem.typegc	0.386	0.940	0.41	0.680	
dem.typeh	-0.565	1.009	-0.56	0.580	
dem.typesg	0.896	0.957	0.94	0.350	
dem.coly	-1.562	0.940	-1.66	0.097	.
dem.sider	-1.078	0.922	-1.17	0.240	
pre.exp1	1.229	0.702	1.75	0.080	.
z.trial	-0.214	0.204	-1.05	0.290	
dem.coly:dem.sider	1.271	1.350	0.94	0.350	

Coxme Model (censor=plug.rem): Number of observations: 264, groups: pig.id, 40
Full Model: plug.rem.L ~ test + z.trial + (1 + z.trial | pig.id)
Null Model: plug.rem.L ~ z.trial + (1 + z.trial | pig.id)

Table S15c. Analysis of Deviance Table

Model	loglik	Chisq	Df	P(>|Chi|)	Sign.
Null	-290.98				
Full	-254.41	73.149	1	<2.2E-16	***

Table S15d. Model Coefficients

	Coefficient	SE	z value	Pr(>|z|)	Sign.
testObserver	4.078	0.638	6.39	1.60E-10	***
z.trial	-0.067	0.155	-0.43	0.67	

Table S16 a-d: Success (Trials 1-5)

GLMM: Number of observations: 155, groups: pig.id, 36
Full Model: success ~ dem.type + dem.col * dem.side + pre.exp + z.trial + (1 + z.trial || pig.id)
Null Model: success ~ dem.col * dem.side + pre.exp + z.trial + (1 + z.trial || pig.id)

Table S16a. Analysis of Deviance Table

Model	npar	AIC	BIC	logLik	deviance	Chisq	Df	Pr(>Chisq)	Sign.
Null	8	96.805	121.15	-40.403	80.805				
Full	11	102.661	136.14	-40.331	80.661	0.144	3	0.986	

Table S16b. Model Coefficients

	Estimate	SE	z value	Pr(>|z|)	Sign.
(Intercept)	-8.977	4.154	-2.161	0.031	*
dem.typeCh	-0.979	4.488	-0.218	0.827	
dem.typeCgc	0.444	4.270	0.104	0.917	
dem.typeCsg	-0.181	3.956	-0.046	0.964	
dem.coly	-1.458	4.253	-0.343	0.732	
dem.sider	-1.386	4.151	-0.334	0.738	
pre.exp1	1.830	2.939	0.623	0.534	
z.trial	-0.467	0.849	-0.550	0.582	
dem.coly:dem.sider	1.812	5.586	0.324	0.746	

GLMM: Number of observations: 264, groups: pig.id, 40
Full Model: success ~ test + z.trial + (1 + z.trial | pig.id)
Null Model: success ~ z.trial + (1 + z.trial | pig.id)

Table S16c. Analysis of Deviance Table

Model	npar	AIC	BIC	logLik	deviance	Chisq	Df	Pr(>Chisq)	Sign.
Null	5	180.25	198.13	-85.128	170.25				
Full	6	117.46	138.91	-52.729	105.46	64.798	1	8.30E-16	***

Table S16d. Model Coefficients

	Estimate	SE	z value	Pr(>|z|)	Sign.
(Intercept)	-21.351	4.098	-5.21	1.89E-07	***
testObserver	11.715	3.074	3.812	0.0001	***
z.trial	-0.486	1.387	-0.351	0.726	

Table S17 a-c: Success (Trial 1)

GLM: Number of observations: 36
Full Model: success ~ dem.type

Table S17a. Single term deletions

	Df	Deviance	AIC	LRT	Pr(>Chi)	Sign.
<none>		36.806	44.806			
dem.type	3	38.139	40.139	1.3332	0.7213	

Table S17b. Model Coefficients

	Estimate	SE	z value	Pr(>|z|)	Sign.
(Intercept)	-1.25	0.80	-1.562	0.118	
dem.typegc	0.56	1.07	0.523	0.601	
dem.typeh	-0.83	1.33	-0.622	0.534	
dem.typesg	0.00	1.13	0	1	

Table S17c. Fisher's Exact Test for Count Data
Number of observations: 58

	p-value	Sign.
success ~ test	0.019	*

Table S18 a-d: Latency to success (Trials 1-5)

Coxme Model (censor=success): Number of observations: 155, groups: pig.id, 36
Full Model: success.L ~ dem.type + dem.col * dem.side + pre.exp + z.trial + (1 + z.trial | pig.id)
Null Model: success.L ~ dem.col * dem.side + pre.exp + z.trial + (1 + z.trial | pig.id)

Table S18a. Analysis of Deviance Table

Model	loglik	Chisq	Df	P(>|Chi|)	Sign.
Null	-126.03				
Full	-125.04	1.981	3	0.576	

Table S18b. Model Coefficients

	Coefficient	SE	z value	Pr(>|z|)	Sign.
dem.typeCh	-0.268	1.890	-0.14	0.89	
dem.typeCgc	1.302	1.813	0.72	0.47	
dem.typeCsg	1.683	1.735	0.97	0.33	
dem.coly	-3.132	1.411	-2.22	0.026	*
dem.sider	-4.391	1.941	-2.26	0.024	*
pre.exp1	3.320	1.439	2.31	0.021	*
z.trial	-0.733	0.374	-1.96	0.05	*
dem.coly:dem.sider	4.982	2.690	1.85	0.064	.

Coxme Model (censor=success): Number of observations: 264, groups: pig.id, 40
Full Model: success.L ~ test + z.trial + (1 + z.trial | pig.id)
Null Model: success.L ~ z.trial + (1 + z.trial | pig.id)

Table S18c. Analysis of Deviance Table

Model	loglik	Chisq	Df	P(>|Chi|)	Sign.
Null	-187.75				
Full	-161.35	52.793	1	3.71E-13	***

Table S18d. Model Coefficients

	Coefficient	SE	z value	Pr(>|z|)	Sign.
testObserver	4.455	0.931	4.78	1.70E-06	***
z.trial	-0.137	0.197	-0.7	0.49	
